# *In vivo* Assessment of Cold Atmospheric Pressure Plasma Technology on the Bioactivity of *Spirulina*

**DOI:** 10.3389/fmicb.2021.781871

**Published:** 2022-01-24

**Authors:** María Consuelo Pina-Pérez, María Úbeda-Manzanaro, Michael Beyrer, Antonio Martínez, Dolores Rodrigo

**Affiliations:** ^1^Departamento de Microbiología y Ecología, Universitat de València, Burjassot, Valencia, Spain; ^2^Food Engineering Laboratory, Institute of Life Technologies, University of Applied Sciences and Arts Western-Switzerland (HES-SO) Valais-Wallis, Sion, Switzerland; ^3^Departamento Conservación y Calidad, Instituto de Agroquímica y Tecnología de Alimentos IATA - Consejo Superior de Investigaciones Científicas (CSIC), Valencia, Spain

**Keywords:** *Spirulina* (*Arthospira*) *platensis*, cold atmospheric plasma (CAP), *in vivo* studies, *C. elegans*, antimicrobial activity, antioxidant activity

## Abstract

The present study challenges the *in vivo* assessment of cold atmospheric pressure plasma (CAPP) technology on the bioactive activity (antioxidant/antiaging and antimicrobial potential) of *Spirulina* powder, using *Caenorhabditis elegans* as an animal model. Surface microdischarge cold atmospheric pressure plasma (SMD-CAPP) treatment was 3.3 W discharge power for 7 min. *C. elegans* lifespan and egg laying were used as indicators of antioxidant/antiaging potential of *Spirulina* (1 mg/mL), when grown with *Spirulina* CP-treated [E_SCP] and untreated [E_S], compared with a control [E_0] (non-supplemented with *Spirulina*). According to our results, under both *Spirulina* supplemented media [E_SCP and E_S] and for the first 17 days, nematodes experienced an increase in lifespan but without significant differences (*p* > 0.05) between control and *Spirulina* CP-treated. Regarding the *in vivo* assay of the antimicrobial potential of *Spirulina* against *Salmonella enterica* serovar Typhimurium (infected worms), no significant differences (*p* > 0.05) were found between the three exposure scenarios (control [S_0]; *Spirulina* supplemented media [S_S]; CP-treated *Spirulina* supplemented media [S_SCP]). According to present results, CAPP-treatment do not influence negatively the lifespan of *C. elegans* but a reduction in the S*pirulina* antiaging potential was found. No *in vivo* modifications in antimicrobial activity seem to be linked to CAPP-processed *Spirulin*a.

## Introduction

In recent years, there has been a steady growth of scientific interest in blue green microalgae *Arthospira platensis*, hereafter referred to as *Spirulina*, as a sustainable source of proteins and other high-value compounds with physiological properties (Ozdemir et al., 2004; Khan et al., 2005, 2006; Nuhu, 2013). *Spirulina* platensis, and its purified extracts, have demonstrated functional properties, of which the most relevant include potential antioxidant, antimicrobial, anti-cancer, and immunomodulatory properties (El-Baky et al., 2008; Nuhu, 2013; Marco Castro et al., 2019). Specifically, the antimicrobial potential of *Spirulina* has been attributed to intracellular and extracellular synthetized metabolites, such as terpenols, sterols, polysaccharides, di-butenolides, peptides, and proteins, secreted by or present in *Spirulina* with demonstrated bactericidal and bacteriostatic effects against clinical and foodborne human pathogens (Lee et al., 2014; Pina-Pérez et al., 2017; Seghiri et al., 2019). The antimicrobial capacity of these *Spirulina* bioactive molecules is currently being exploited in useful applications by the food industry, and pharmacological and cosmetic sectors (Grahl et al., 2018; Martelli et al., 2020).

One of the most pertinent aspects of novel *Spirulina*-derived product formulation is related with the stability/functionality maintenance of bioactive molecules after processing (proteins, peptides, lipids, polyphenols, polysaccharides) (Chaiklahan et al., 2012; Pina-Pérez et al., 2018; Cassani et al., 2020). Recent trends are moving toward more efficient, environmentally friendly, and very rapid non-thermal treatments (ranging from a few seconds to a few minutes), implemented in the food and pharmaceutical industries to inactivate pathogenic bacteria, while preserving the techno-functional product properties intact (flavor, color, texture, solubility) as well as the beneficial bioactive potential in the end product. Among these novel processes, cold plasma (CP) is emerging as a non-thermal technology, with huge versatility to treat solid, liquid and powder-based matrices, proving effective in the inactivation of spores, viruses, mycotoxins and prions (Elmoualij et al., 2012; ten Bosch et al., 2017; Beyrer et al., 2020; Pina-Pérez et al., 2020). Plasma has been defined as the fourth state of matter, a partially ionized gas (Helium, Argon, air, other mixtures) with unique properties. Radio-frequency, microwaves, thermal energy, electric and magnetic fields [plasma jet, surface microdischarge plasma (SMD), dielectric barrier discharge plasma (DBD)] are used as energy sources for gas ignition, with the generated plasma being comprised by ions (positive and negative), free electrons, radicals, and electromagnetic radiation (photons UV and visible light) (Tolouie et al., 2018). To date, the continuous generation of electrical microdischarges is the most efficient method of choice to generate cold plasma (temperature 30–40°C) (Pina-Pérez et al., 2020).

In spite of the promising results obtained so far in terms of the microbiological safety obtained by CP application, some effects still remain unknown (Whitehead, 2016; Ziuzina and Misra, 2016). In fact, the complex chemistry generated by plasma ignition (hydroxyl radicals (OH), hydrogen peroxide (H_2_O_2_), ozone (O_3_), superoxide anion radicals (•O^2–^), atomic oxygen (O), nitric oxide (NO), nitrite/nitrate (NO_2_^–^/NO_3_^–^), and subsequently, the dynamic interaction of plasma reactive species with food macromolecules requires [food-plasma treatment] in-depth evaluation. These mainly concern the bioactive structure (side-toxic compounds) and functionality (antioxidant or antimicrobial activity, among others) after processing (Sarangapani et al., 2017; Pérez-Andrés et al., 2019). To date, very few studies (mainly focused on lipids oxidation and proteins denaturation) have been published regarding potential biological risks associated to cold plasma when used in food/pharmaceutical processing (for instance regarding the functional effect of these novel CP processed matrices) (Gavahian et al., 2018; Alves Filho et al., 2019).

In spite of the rich-nutritional value of the *Spirulina* matrix, including complex polysaccharides, vitamins and polyphenols with antiaging properties (scavenging free radicals, reducing DNA damage, and inhibiting ROS accumulation) and antimicrobial capacity, no previous studies have evaluated *Spirulina* functionality *in vivo* after CAPP processing. Therefore, the present study aims to evaluate the effect of cold atmospheric pressure plasma technology on the antioxidant/antiaging and antimicrobial bioactive potential of *Spirulina* against *Salmonella enterica* serovar Typhimurium, by using *Caenorhabditis elegans* as *in vivo* model.

## Materials and Methods

### *Spirulina* Powder

A *Spirulina* (*Arthrospira platensis*) powder (Spirulina Plus) was purchased from Phytopharma S.A. (Grandvillard, Switzerland).

### Surface Microdischarge Cold Atmospheric Pressure Plasma Treatment

In the present study, a Surface Microdischarge Cold Atmospheric Pressure Plasma (SMD-CAPP) equipment was used fully developed and constructed by the Institute of Systems Engineering (HES-SO Valais-Wallis, Sion, Switzerland). The system is built basically with a high-voltage powered planar, stainless steel grid electrode (total surface area = 149.76 cm^2^; grid size = 9.8 × 9.4 mm^2^), mounted with a dielectric barrier made from Teflon, and a water-cooled ground electrode. Plasma is ignited on air. The distance between the plasma active zone (powered electrode) and the sample is 6 mm. The electric circuit was described before (Pina-Pérez et al., 2020). The CAPP treatment settings were selected according to the demonstrated ≥2 log10 inactivation of *B. subtilis* spores embedded in a starch powder (Beyrer et al., 2020). In short, a discharge power of 3.3 W was applied on a thin layer of *Spirulina* powder homogeneously spread on sterile glass slides (0.5 mg/cm^2^). The treatment time was 7 min and the voltage frequency 10 kHz.

### *Caenorhabditis elegans* Studies

In the present study, the nematode *C. elegans* strain N2 was used, provided by the College of Biological Sciences, Minnesota University, United States. Nematodes were routinely maintained in NGM (Nematode Growth Media) (Stiernagle, 2006) petri dishes, in a bacterial lawn of *E. coli* OP50 (non-infected studies) or *Salmonella* Typhimurium (CECT 443) (infected studies) (Table 1). The worms at larval stage L4 were obtained by synchronization (Sanz-Puig et al., 2020). For lifespan studies, once synchronized, L4 nematodes (initially 250), were periodically transferred to plates (25 plates; 10 nematodes per plate), maintained at 20°C during their life cycle (approximately 3 weeks), and examined at 48 h intervals with a binocular microscope (COMECTA S.A.). Worms were considered dead when they did not move or do not respond to stimulation (contact with a platinum worm picker).

**TABLE 1:** Groups studied and feeding media.

	Group	Feeding media^a^
Non-infected	Control [E_0]	*E. coli* OP50
	*Spirulina* [E_S]	*E. coli* OP50 + 1 mg/mL non-treated *Spirulina*
	*Spirulina* CP-treated [E_SCP]	*E. coli* OP50 + 1 mg/mL CP-treated *Spirulina*
Infected	Control [S_0]	*S.* Typhimurium
	*Spirulina* [S_S]	*S*. Typhimurium + 1 mg/mL non-treated *Spirulina*
	*Spirulina* CP-treated [S_SCP]	*S*. Typhimurium + 1 mg/mL CP-Treated *Spirulina*

*^a^Nematodes belonging to all groups were maintain in NGM agar with the addition of the different microorganism cultures and Spirulina, depending on the study group.*

For nematode egg laying studies, 25 adult worms were distributed in 25 plates per substrate under study. Plates were incubated at 20^°^C for 48 or 72 h. After this time, the progeny (eggs and larvae) of each adult worm was counted and the worm was transferred to a new plate of the same substrate. The procedure was repeating until the nematode’s death.

*Caenorhabditis elegans* lifespan and egg-laying capacity were recorded for the different study and control groups (Table 1). For non-infected assays, NGM plates with non-CP treated *Spirulina* [E_S] and CP-treated *Spirulina*, [E_SCP], were seeded with a bacterial lawn of *E. coli* OP50. For infected studies, plates with non-CP treated *Spirulina* [S_S] and CP-treated *Spirulina* [S_CPS] were seeded with a *S*. Typhimurium bacterial lawn to simulate infection Mathematical modeling and statistical analysis.

The Weibull distribution function was fit to the survival curves (Mafart et al., 2002) (Equation 1). To do so, Ginafit software was used (Geeraerd et al., 2005).


(1) $$Log_{10}\left(N\right)=Log_{10}\left({\left(\frac{t}{\delta }\right)}^{p}\right)$$

where N is the number of alive worms at time t, N_0_ is the number of worm population at time zero (t_0_), ð is the kinetic parameter (days/worms) and represents the time of first decimal reduction for a specific worm and, p is the shape parameter of the Weibull distribution function.

Kaplan–Meirer estimator (Kaplan and Meier, 1958) was also used to fit survival experimental data as a function of time.


(2) $$S\left(t\right)=\prod_{t_{i\leq t}}\left(1-\frac{d_{i}}{n_{i}}\right)$$

Where, t_*i*_ is a time when at least one event happened, d_*i*_ is the number of events that happened at time t_*i*_, and n_*i*_ represents the number of individuals known to have survived up to time t_*i*_.

Percentiles for survival curves were obtained for each experimental group, and ANOVA analysis were performed to determine significant differences between groups. Statgraphics Centurion XVII software was used for these analyses.

## Results

### Effect of Cold Atmospheric Pressure Plasma Treatment of *Spirulina* on the Lifespan and Reproductive-Rate of *Caenorhabditis elegans* Populations

**FIGURE 1:**
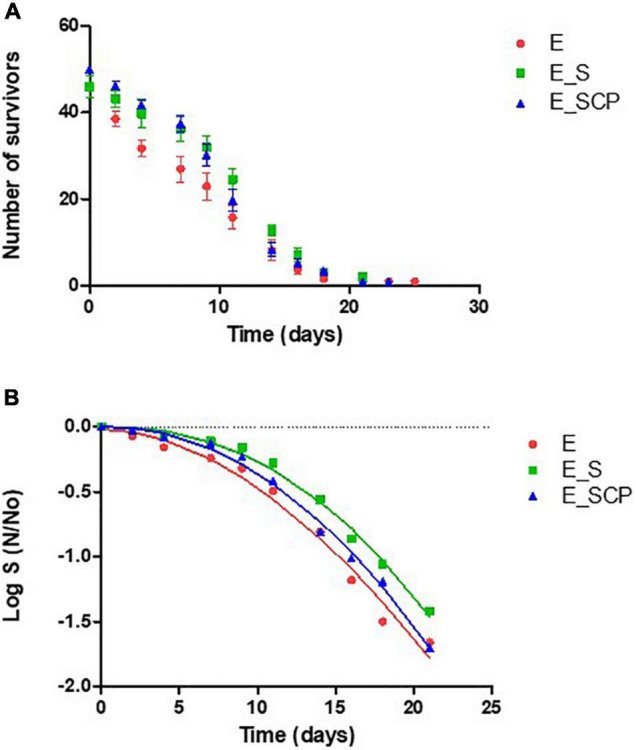
**(A)** Survival curves for non-infected worms fed on different substrates. **(B)** Fitted survivor data to Weibull model. NGM without a supplement (control sample) [E_0]; NGM supplemented with non-treated *Spirulina* [E_S]; and NGM supplemented with CP-treated *Spirulina* [E_SCP].

The lifespan assay is an index used to evaluate the bioactive potential (antiaging effect) of different compounds (Ayyadevara et al., 2013; Jattujan et al., 2018; Chen et al., 2020; Ibáñez-Peinado et al., 2020). The cumulative survival curves of the nematode population fed with the different substrates can be seen in Figure 1. It clearly shows that the number of live worms decreased over time until approximately 21 days for all populations studied. In the case of the nematode population exposed to *Spirulina* [E_S] and [E_SCP], lower nematode death rates per time interval compared to control samples appear, probably due to a protective antiaging effect due to *Spirulina* exposure, regardless of whether the *Spirulina* had been treated with cold plasma [E_SCP] or not [E_S].

Survival and censored data for each time interval were analyzed with the Kaplan Meier estimator in order to obtain the percentile tables indicating the number of nematodes that survive up to a certain time point (Table 2). Significant differences on the lifespan of the control and nematodes fed with untreated *Spirulina* supplement (*p* ≤ 0.05) were determined for all percentiles (75, 50, and 25). Focusing on the 50th percentile, 50% of the population fed with untreated *Spirulina* [E_S] survive until day 12, meanwhile, in the control substrate [E_0], nematodes remain alive only until day 9, approximately. These results confirm what was previously mentioned for the cumulative survival curves. The lifespan of nematodes fed with a supplement of CP-treated *Spirulina* is in between the control and group fed with the supplement of non-treated *Spirulina*, specifically, values are higher than those reported for [E_0] but lower than the ones reported for [E_S]; however, values showed non-significant differences (*p* > 0.05) between either of the two. These findings are, probably, in concordance with previous results showing that the CP treatment applied was able to inactivate at least 2 log10 of *B. subtilis* spores embedded in the *Spirulina* powder but, at the same time, a decrease in nutritive value, measured as a reduction in total phenolic compounds (TPC) and antioxidant activity (TEAC), was found for *Spirulina* CP treated samples (Beyrer et al., 2020).

**TABLE 2:** Lifespan (days) of non-infected nematodes for the different percentile depending on group studied.

Percentile of survivors [%]	Lifespan of nematodes [d]		
	Control [E_0]	Untreated *Spirulina* [E_S]	CP treated *Spirulina* [E_SCP]
75	5.20 ± 0.42^a^	7.83 ± 0.70^b^	7.26 ± 0.76^ab^
50	8.79 ± 0.98^a^	12.27 ± 0.75^b^	10.16 ± 0.81^ab^
25	12.33 ± 0.80^a^	15.41 ± 0.67^b^	12.93 ± 0.73^a^

*^a–b^Letter superscripts are indicating significant differences (p ≤ 0.05) between rows.*

The parameters of the fitted Weibull function for observed survivors (Figure 1) are given in Table 3. The group of nematodes fed with a *Spirulina* supplemented NGM [E_S] present higher value of ð (time to failure) (17.24 ± 0.21 vs. 15.14 ± 0.28 for *spirulina* supplemented and control sample, respectively), which means that the death rate appears to be lower than that of the population fed in the absence of *Spirulina*. The kinetic constant ð of the group fed with CP-treated *Spirulina* supplemented NGM () is, again, in between the two other groups but not significantly different from the one or the other (15.14 ± 0.28, 17.24 ± 0.21, and 16.96 ± 1.16 for control, *Spirulina* and CP-treated *Spirulina*, respectively). So, the added nutritional value of CP-treated *Spirulina* CP cannot be confirmed statistically, which is in line with predictions with the Kaplan-Meier estimator. Consequently, the results showed that CP-treatment do not influence negatively the lifespan of *C. elegans* but a reduction in the *Spirulina* antiaging potential was found.

**TABLE 3:** Fit parameters of the survival curves (Weibull distribution function) for non-infected *C. elegans* populations fed with different substrates.

	Kinetic constant—ð [worms/d]	Shape parameter *p* [-]	R^2^_adjusted_ [-]	RMSE [-]
Control [E_0]	15.14 ± 0.28^a^	1.96 ± 0.25	0.98	0.034
*Spirulina* [E_S]	17.24 ± 0.21^b^	2.73 ± 0.19	0.99	0.058
*Spirulina* CP-treated [E_SCP]	16.96 ± 1.16^ab^	2.12 ± 0.36	0.91	0.046

*^a–b^Different subscripts in the column indicate significant differences for ð.*

**FIGURE 2:**
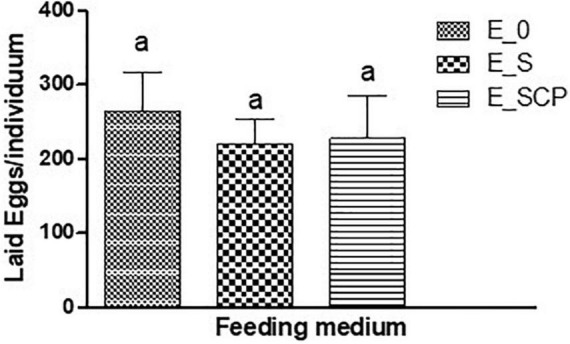
Effect of feeding substrate on average egg laying per nematode of *E. elegans*: NGM without a supplement (control sample) [E_0]; NGM supplemented with non-treated *Spirulina* [E_S]; and NGM supplemented with CP-treated *Spirulina* [E_SCP].

The *C. elegans* egg laying pattern is considered another valuable indicator of the *in vivo* impact associated to natural bioactivity, toxin exposure, and evaluation of other chemical-mediated disorders (Nidheesh et al., 2016; Teshiba et al., 2016; Peixoto et al., 2017; Salgueiro et al., 2017). Figure 2 shows the total number of eggs laid by each worm throughout its fertile phase. Nominally, the worms fed on the control substrate [E_0] laid more total eggs per individual than those fed on the substrate supplemented with CP-treated [E_SCP], or untreated [E_S] *Spirulina* powder. Statistically the differences were non-significant and a negative effect of *Spirulina* powder, CP treated or not, on egg laying of *C. elegans* cannot be concluded.

### Effect of Cold Atmospheric Pressure Plasma on the Antimicrobial Activity of *Spirulina* Assessed on *Salmonella* Typhimurium Infected Worms

*Caenorhabditis elegans* has been used very frequently as a model of pathogenesis, and in particular for infection by pathogenic microorganisms (Balla and Troemel, 2013; Curt et al., 2014). In this sense, there are published studies in which the antimicrobial activity of a compound has been determined based on a higher survival of infected *C. elegans* when exposed to the antimicrobial compounds when compared with infected control nematodes (non-exposed to the antimicrobial compound) (Sanz-Puig et al., 2017; Ibáñez-Peinado et al., 2020; Palacios-Gorba et al., 2020). In the present study, an independent set of experiments was specifically designed to study the *Spirulina* (treated and untreated by CP) antimicrobial activity against *Salmonella enterica* serovar Typhimurium when *C. elegans* worms were infected.

**FIGURE 3:**
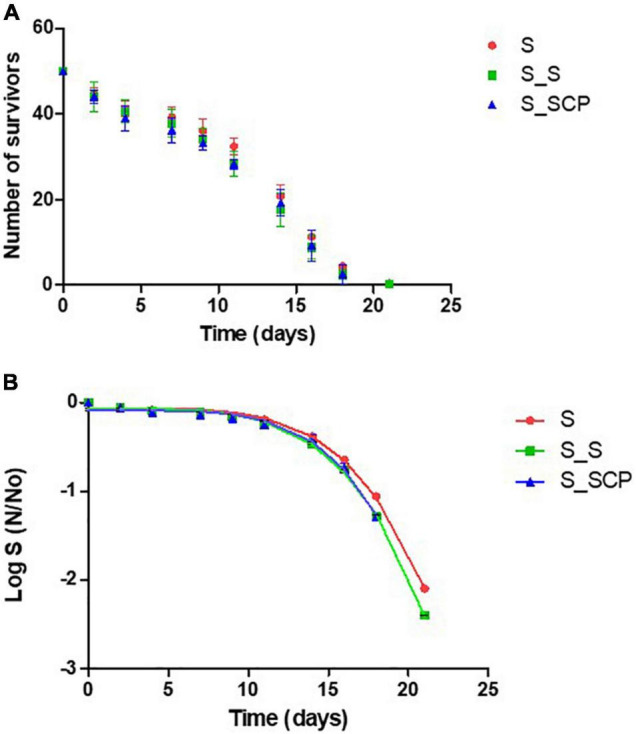
**(A)** Survival curves for worms infected with *S*. Typhimurium and cultivated on different substrates. **(B)** Fitted survivor data to Weibull model. NGM without a supplement (control sample) [S_0]; NGM supplemented with non-treated *Spirulina* [S_S]; and NGM supplemented with CP-treated *Spirulina* [S_SCP].

Figure 3 shows the survival curves for the nematodes infected with *S*. Typhimurium and grown on a non-supplemented substrate [S_0] that will be used as a control, and on a substrate supplemented with untreated [S_S] and CP-treated *Spirulina* [S_SCP]. No significant differences on survival (neither changes in lifespan) (*p* > 0.05) of either non-treated or treated *Spirulina* with control sample was observed. Consequently, no reduction in infection was detected when infected nematodes were exposed to *Spirulina*, either untreated or treated by cold plasma.

In the same sense, data mining with the Kaplan-Meier estimation for computing the lifespan (Table 4) and fit parameters in the Weibull model for simulating the survival curve (Table 5), do not indicate differences between control samples [S_0] (nematodes fed without *Spirulina* supplementation) nor between nematodes with *Spirulina* supplementation when considering the impact of the plasma treatment [S_S] and [S_SCP]. The lifespan for percentiles (75, 50, and 25%) of the population and kinetic constants ð in the Weibull model do not differ significantly for different substrates. Therefore, it can be concluded that the presence of *Spirulina*, treated or not by CAP, does not modify the survival of *C. elegans*, therefore, *Spirulina* does not present antimicrobial capacity *in vivo* against *S*. Typhimurium if it is determined by a reduced pathogenicity.

**TABLE 4:** Lifespan (days) of infected nematodes for the different percentile depending on group studied.

Percentile of survivors [%]	Lifespan of nematodes [d]		
	Control [S_0]	Untreated *Spirulina* [S_S]	CP treated *Spirulina* [S_SCP]
75	8.53 ± 1.25 ^a^	6.99 ± 1.36 ^a^	5.88 ± 1.98 ^a^
50	13.07 ± 0.39 ^a^	12.07 ± 0.79 ^a^	12.25 ± 0.47 ^a^
25	15.9 ± 0.35 ^a^	15.20 ± 0.64 ^a^	15.47 ± 0.67 ^a^

*^a^Letter superscript indicate significant differences (p ≤ 0.05) between rows.*

**TABLE 5:** Fit parameters of the survival curves (Weibull distribution function) for infected *C. elegans* populations fed with different substrates.

	Kinetic constant ð [worms/d]	Shape parameter *p [*-*]*	R^2^_adjusted_ [-]	RMSE [-]
Control [S_0]	18.00 ± 0.31^a^	4.58 ± 0.16	0.99	0.032
*Spirulina* [S_S]	17.28 ± 0.23^a^	4.34 ± 0.16	0.99	0.039
*Spirulina* CP-treated [S_SCP]	17.38 ± 0.37^a^	4.75 ± 0.53	0.98	0.053

*^a^Different subscripts in the column indicate significant differences for ð.*

## Discussion

*Spirulina* has been incorporated as an ingredient of many recently launched food products (2015–2020: dairy products—cheese, yogurt, smoothies; bakery products—cookies, bread, snacks; pasta; sauces, among others). This whole ingredient and its purified bioactive compounds are mostly commercialized and employed in powder form, to make up the final product, not only in the food industry but also in the pharmaceutical sector (development of novel nutraceutical products). These innovative matrices are subjected to different conventional and novel processing technologies (drying, lyophilization, roasting, thermal sterilization, extrusion, homogenization, fermentation) (Caporgno and Mathys, 2018), which can affect some macromolecules in the *Spirulina* processed matrix. Proteins and lipids in foods are prone to oxidation during industrial processing or storage; essential nutrients may be broken down and potentially toxic compounds might be generated (e.g., formation of 5-hydroxymethylfurfural and reduction of antioxidant activity after thermal treatment) (Kowalski, 2013; Winkler-Moser et al., 2020). Despite the positive antioxidant activity associated to *Spirulina* microalgae reported in the scientific literature, very few studies have evaluated the impact of novel processing technologies on this highly nutritious matrix (Colla et al., 2017; Beyrer et al., 2020). Indeed, it is important to gain greater insight into how these novel processes can degrade or affect the most unstable and valuable compounds (lipids, phycocyanin, polysaccharides, peptides, or vitamins) of *Spirulina* (Agustini et al., 2015; Caporgno and Mathys, 2018; Beyrer et al., 2020).

As for cold plasma (CP) as an innovative processing non-thermal technology, very few studies to date have reflected the physical-chemical analysis of CP processed food matrices, explaining the impact of complex plasma chemistry on food components (Bußler et al., 2016; Pankaj et al., 2018). Degradation of polysaccharides, loss of color, vitamin content, and total phenolic compounds, jointly with lipid oxidation have scarcely been described *in vitro*, with strong dependence on the type of plasma applied, gas carrier, and treatment intensity used (Grzegorzewski et al., 2011; Kim et al., 2013; Rodríguez et al., 2017). Han et al. (2016) studied the *in vivo* effect of CP food processed matrices assessing possible associated risks. Results revealed no acute toxicity associated to CP-treated soy-based edible films assessed in a rat model (Han et al., 2016). However, to date there are hardly any studies published on cold plasma effects of food bioactivity *in vivo*.

According to our results, it seems that no-negative effect is derived from SMD-CAPP treatment of *Spirulina* (3.3 W power discharge; 7 min) that could diminish the survival of *C. elegans* worms; however, it cannot be concluded the same bioactive/antiaging properties of the processed product as shown by *in vivo* assays in the *C. elegans* model. Previous studies have demonstrated the potential of *C. elegans* to test antioxidant/anti-aging *in vivo* properties associated to other natural compounds, such as green tea, purple wheat or açai (*Euterpe precatoria* Mart.) (Chen et al., 2013; Abbas and Wink, 2014; Peixoto et al., 2016). This animal model was also used by Wilson et al. (2010) to evaluate the bioactivity of blueberry proanthocyanins and a collection of resveratrol analogs, concluding that these phytochemicals enhance longevity and stress resistance of *C. elegans* adult worms. More recently, Ibáñez-Peinado et al. (2020) studied the effect of a cauliflower extract on nematode lifespan in a population fed on enriched media. Results indicated that the cauliflower extract had a protective effect on nematodes senescence. Authors reported a percentile 50 of 12.4 days, representing a significant increase in comparison with the 6.92 days for nematodes lifespan when fed on the substrate not supplemented with cauliflower extract (NMG). The results obtained by Ibáñez-Peinado et al. (2020) are similar to those obtained in the present work for the same percentile 50th, with *C. elegans* lifespan being increased significantly when fed on *Spirulina* (untreated) supplemented substrates (12.27 vs. 8.79 days in NMG non supplemented). As indicated by Ibáñez-Peinado et al. (2020) and also by Fang et al. (2019), it is likely that natural extracts from plants and, in this case from algae, would produce an antioxidant effect by means the up-regulating the expression of antioxidant-related genes in *C. elegans*, and inhibiting cell apoptosis, improving the nematode antioxidant defense system, leading to the lengthening of the lifespan.

With respect to microalgae antioxidants, Beyrer et al. (2020) demonstrated that no significant differences (*p* > 0.05) were detected on total phenolic content (TPC) of treated *Spirulina* powder (SMD-CAPP ignited on air range 1.1–2.2 W discharge power), however, the antioxidant potential attributed to *Spirulina* powder was slightly reduced when exposed to that specific treatment. This reduction seems to be related with the slight reduction in the *in vivo* antiaging potential of *Spirulina* described in the present manuscript, although non-significant differences have been detected between nematodes lifespan when fed with untreated vs. CAPP treated *Spirulina*, even under CAPP ignited on air—3.3 W treatments, applied at longer treatment times (7 min).

In relation to the antimicrobial bioactive potential of *Spirulina*, it has been extensively reported *in vitro* (Al-Ghanayem, 2017; Pina-Pérez et al., 2018). To date, *C. elegans* has been used as *in vivo* model for novel antimicrobial drug research, to study therapies against *Staphylococcus aureus*, *Pseudomonas aeruginosa*, *Enterococcus faecalis*, and *H. pylori* (Kong et al., 2016). The present results are the first to provide an *in vivo* approach to evaluate the antimicrobial potential of *Spirulina* powder (1 mg/mL) against *Salmonella enterica* serovar Typhimurium. However, no significant antimicrobial activity was detected *in vivo* due to *C. elegans* exposure to the *Spirulina* concentration assayed (1 mg/mL). Neither did the air ignited cold plasma provided any positive or negative antimicrobial effect on infected *C. elegans* worms, with similar lifespan rates as those in control samples without *Spirulina*. Other algae compounds have demonstrated antimicrobial potential *in vivo* using the *C. elegans* animal model (Palacios-Gorba et al., 2020). According to these authors, fucoidan (a sulphated polysaccharide from Phaeophyceae) demonstrates potential antimicrobial activity *in vivo* against *Helicobacter pylori*, when administered in the range 50–200 μg/ml. Also, Ibáñez-Peinado et al. (2020) demonstrated that a cauliflower extract increased the survival of a *C. elegans* population infected with *Salmonella enterica*, obtaining 50th percentile values equivalent to 8.8 days in supplemented substrate (NMG+cauliflower), in relation to 50th percentile of 4.4 days for a nematode population infected and fed on NMG plates (not supplemented).

The present study is the first to provide mathematical modeling of *C. elegans* survival under *Spirulina* exposure (CP-treated and untreated), via use of the Weibull distribution function. The value of p (shape parameter) has a marked effect on the failure rate (worm death rate) of the Weibull distribution. Inferences can be drawn about a population’s failure characteristics (worm death rate) by considering whether the value of p is less than, equal to, or greater than one. If *p* < 1, the model exhibits a failure rate that decreases with time, in populations where *p* = 1 there is a constant failure rate (consistent with the exponential distribution), and populations with *p* > 1 have a failure rate that increases with time, as in the case of this study. Probably this behavior is linked to the age of worms and reflects the senescence process. At the same time, the Weibull distribution function provides kinetic parameters to objectively describe survival curves of exposed nematodes, either uninfected or infected by *Salmonella* and exposed to CP-treated *Spirulina*, and quantify worm death rate under different study conditions.

## Conclusion

Surface Microdischarge Cold Atmospheric Pressure Plasma is currently considered an effective new technology to sterilize food and pharmaceutical matrices, in a few minutes (<7 min), when plasma is ignited on air (cost-effective process). However, further research is required in relation to food macromolecules stability/functionality under such new treatments, mainly considering the dynamic chemistry generated during food-plasma interaction. According to the results of the present study, no negative effects were recorded on *C. elegans* indicators, such as lifespan and reproductive rate after SMD-CAPP treatment (no reduction was detected). Validation of *Spirulina* bioactivity (i.e., improved *C. elegans* lifespan) has been demonstrated and mathematically modeled for the first time. No antimicrobial effect, measured as an increase in *C. elegans* lifespan, was detected in worms infected with *Salmonella enterica* serovar Typhimurium when *Spirulina* was added to the media a concentration of 1 mg/ml.

## Data Availability Statement

The raw data supporting the conclusions of this article will be made available by the authors, without undue reservation.

## Author Contributions

DR and AM: conceptualization. DR, MÚ-M, and MP-P: methodology. MÚ-M and MP-P: experimental work. MP-P, AM, DR, and MB: writing—original draft preparation, writing—review and editing, and funding acquisition. All authors have read and agreed to the published version of the manuscript.

## Conflict of Interest

The authors declare that the research was conducted in the absence of any commercial or financial relationships that could be construed as a potential conflict of interest.

## Publisher’s Note

All claims expressed in this article are solely those of the authors and do not necessarily represent those of their affiliated organizations, or those of the publisher, the editors and the reviewers. Any product that may be evaluated in this article, or claim that may be made by its manufacturer, is not guaranteed or endorsed by the publisher.

## References

[B1] Abbas S. Wink M. (2014). Green tea extract induces the resistance of *Caenorhabditis elegans* against oxidative stress. *Antioxidants* 3 129–143. 10.3390/antiox3010129 26784668 PMC4665450

[B2] Agustini T. W. Suzery M. Sutrisnanto D. (2015). Comparative study of bioactive substances extracted from fresh and dried *Spirulina* sp. *Procedia Environ. Sci.* 23 282–289. 10.1016/j.proenv.2015.01.042

[B3] Al-Ghanayem A. A. (2017). Antimicrobial activity of *Spirulina platensis* extracts against certain pathogenic bacteria and fungi. *Adv. Biores.* 8 96–101. 10.15515/abr.0976-4585.8.6.96101

[B4] Alves Filho E. G. Silva L. M. A. Filho F. O. Rodrigues S. Fernandes F. A. N. Gallão M. I. (2019). Cold plasma processing effect on cashew nuts composition and allergenicity. *Food Res. Int.* 125 1–9. 10.1016/j.foodres.2019.108621 31554108

[B5] Ayyadevara S. Bharill P. Dandapat A. Hu C. Khaidakov M. Mitra S. (2013). Aspirin inhibits oxidant stress, reduces age-associated functional declines, and extends lifespan of *Caenorhabditis elegans*. *Antiox. Redox Signal.* 18 481–490. 10.1089/ars.2011.4151 22866967

[B6] Balla K. M. Troemel E. R. (2013). *Caenorhabditis elegans* as a model for intracellular pathogen infection. *Cell. Microbiol.* 15 1313–1322. 10.1111/cmi.12152 23617769 PMC3894601

[B7] Beyrer M. Pina-Perez M. C. Martinet D. Andlauer W. (2020). Cold plasma processing of powdered Spirulina algae for spore inactivation and preservation of bioactive compounds. *Food Control* 118 1–8. 10.1016/j.foodcont.2020.107378

[B8] Bußler S. Rumpold B. A. Fröhling A. Jander E. Rawel H. M. Schlüter O. K. (2016). Cold atmospheric pressure plasma processing of insect flour from *Tenebrio molitor*: impact on microbial load and quality attributes in comparison to dry heat treatment. *Innov. Food Sci. Emerg. Technol.* 36 277–286. 10.1016/j.ifset.2016.07.002

[B9] Caporgno M. P. Mathys A. (2018). Trends in microalgae incorporation into innovative food products with potential health benefits. *Front. Nutr.* 5:58. 10.3389/fnut.2018.00058 30109233 PMC6080594

[B10] Cassani L. Gomez-Zavaglia A. Jimenez-López C. Lourenço-Lopes C. Prieto M. A. Simal-Gandara J. (2020). Seaweed-based natural ingredients: stability of phlorotannins during extraction, storage, passage through the gastrointestinal tract and potential incorporation into functional foods. *Food Res. Int.* 137 1–14. 10.1016/j.foodres.2020.109676 33233253

[B11] Chaiklahan R. Chirasuwan N. Bunnag B. (2012). Stability of phycocyanin extracted from *Spirulina* sp.: influence of temperature, pH and preservatives. *Process. Biochem.* 47 659–664. 10.1016/j.procbio.2012.01.010

[B12] Chen W. Müller D. Richling E. Wink M. (2013). Anthocyanin-rich purple wheat prolongs the life span of *Caenorhabditis elegans* probably by activating the daf-16/foxo transcription factor. *J. Agric. Food Chem.* 61 3047–3053. 10.1021/jf3054643 23470220

[B13] Chen Y. Wan X. Wu D. Ouyang Y. Gao L. Chen Z. (2020). Characterization of the structure and analysis of the anti-oxidant effect of microalga *Spirulina platensis* polysaccharide on *Caenorhabditis elegans* mediated by modulating microRNAs and gut microbiota. *Int. J. Biol. Macromol.* 163 2295–2305. 10.1016/j.ijbiomac.2020.09.041 32931825

[B14] Colla L. M. Bertol C. D. Ferreira D. J. Bavaresco J. Costa J. A. V. Bertolin T. E. (2017). Thermal and photo-stability of the antioxidant potential of *Spirulina platensis* powder. *Braz. J. Biol.* 77 332–339. 10.1590/1519-6984.14315 27683806

[B15] Curt A. Zhang A. Minnerly J. Jia K. (2014). Intestinal autophagy activity is essential for host defense against *Salmonella typhimurium* infection in *Caenorhabditis elegans*. *Dev. Comp. Immunol.* 45 214–218. 10.1016/j.dci.2014.03.009 24674884

[B16] El-Baky H. H. A. El Baz F. K. El-Baroty G. S. (2008). Characterization of nutraceutical compounds in blue green alga *Spirulina maxima*. *J. Med. Plants Res.* 2 292–300. 10.5897/JMPR.9000331

[B17] Elmoualij B. Thellin O. Gofflot S. Heinen E. Levif P. Séguin J. (2012). Decontamination of prions by the flowing afterglow of a reduced-pressure N_2_-O_2_ cold-plasma. *Plasma Proc. Polym.* 9 612–618. 10.1002/ppap.201100194

[B18] Fang Z. Chen Y. Wang G. Feng T. Shen M. Xiao B. (2019). Evaluation of the antioxidant effects of acid hydrolysates from *Auricularia auricular* polysaccharides using a *Caenorhabditis elegans* model. *Food Funct.* 10 5531–5543. 10.1039/C8FO02589D 31418439

[B19] Gavahian M. Chu Y. H. Khaneghah A. M. Barba F. J. Misra N. N. (2018). A critical analysis of the cold plasma induced lipid oxidation in foods. *Trends Food Sci. Technol.* 77 32–41. 10.1016/j.tifs.2018.04.009

[B20] Geeraerd A. H. Valdramidis V. P. Van Impe J. F. (2005). GInaFiT, a freeware tool to assess non-log-linear microbial survivor curves. *Int. J. Food Microbiol.* 102 95–105. 10.1016/j.ijfoodmicro.2004.11.038 15893399

[B21] Grahl S. Strack M. Weinrich R. Mörlein D. (2018). Consumer-Oriented product development: the conceptualization of novel food products based on Spirulina (*Arthospira platensis*) and resulting consumer expectations. *J. Food Qual.* 2018:1919482. 10.1155/2018/1919482

[B22] Grzegorzewski F. Ehlbeck J. Schlüter O. Kroh L. W. Rohn S. (2011). Treating lamb’s lettuce with a cold plasma—influence of atmospheric pressure air plasma immanent species on the phenolic profile of *Valerianella locusta*. *LWT Food Sci. Technol.* 44 2285–2289. 10.1016/j.lwt.2011.05.004

[B23] Han S. H. Suh H. J. Hong K. B. Kim S. Y. Min S. C. (2016). Oral toxicity of cold plasma-treated edible films for food coating. *J. Food Sci.* 81 3052–3057. 10.1111/1750-3841.13551 27861860

[B24] Ibáñez-Peinado D. Pina-Pérez C. García-Carrión G. Martínez A. Rodrigo D. (2020). In vivo antimicrobial activity assessment of a cauliflower by-product extract against *Salmonella typhimurium*. *Front. Sustain. Food Syst.* 4:8. 10.3389/fsufs.2020.00008

[B25] Jattujan P. Chalorak P. Siangcham T. Sangpairoj K. Nobsathian S. Poomtong T. (2018). *Holothuria scabra* extracts possess anti-oxidant activity and promote stress resistance and lifespan extension in *Caenorhabditis elegans*. *Exp. Gerontol.* 110 158–171. 10.1016/j.exger.2018.06.006 29902502

[B26] Kaplan E. L. Meier P. (1958). Nonparametric estimation from incomplete observations. *JASA* 53 457–481.

[B27] Khan M. Shobha C. J. Mohan J. I. Rao U. M. Prayag A. N. Kutala K. V. (2006). Spirulina attenuates cyclosporine-induced nephrotoxicity in rats. *J. Appl. Toxicol.* 26 444–451. 10.1002/jat.1159 16858688

[B28] Khan M. Shobha C. J. Rao U. M. Sundaram C. M. Singh S. Mohan J. I. (2005). Protective effect of Spirulina against doxorubicin-induced cardiotoxicity. *Phytother. Res.* 19 1030–1037. 10.1002/ptr.1783 16372368

[B29] Kim H.-J. Yong H. I. Park S. Choe W. Jo C. (2013). Effects of dielectric barrier discharge plasma on pathogen inactivation and the physicochemical and sensory characteristics of pork loin. *Curr. Appl. Phys.* 13 1420–1425. 10.1016/j.cap.2013.04.021

[B30] Kong C. Eng S. A. Lim M. P. Nathan S. (2016). Beyond traditional antimicrobials: a *Caenorhabditis elegans* model for discovery of novel anti-infectives. *Front. Microbiol.* 7:1956. 10.3389/fmicb.2016.01956 27994583 PMC5133244

[B31] Kowalski S. (2013). Changes of antioxidant activity and formation of 5-hydroxymethylfurfural in honey during thermal and microwave processing. *Food Chem.* 15 1378–1382. 10.1016/j.foodchem.2013.04.025 23790927

[B32] Lee S.-Y. Kim K.-B.-W.-R. Lim S. Ahn D.-H. (2014). Antibacterial mechanism of *Myagropsis myagroides* extract on *Listeria monocytogenes*. *Food Control* 42 23–28. 10.1016/j.foodcont.2014.01.030

[B33] Mafart P. Couvert O. Gaillard S. Leguerinel I. (2002). On calculating sterility in thermal preservation methods: application of the Weibull frequency distribution model. *Int. J. Food Microbiol.* 72 107–113. 10.1016/S0168-1605(01)00624-9 11843401

[B34] Marco Castro E. Shannon E. Abu-Ghannam N. (2019). Effect of fermentation on enhancing the nutraceutical properties of *Arthospira platensis* (Spirulina). *Ferment.* 5 1–19. 10.3390/fermentation5010028

[B35] Martelli F. Cirlini M. Lazzi C. Neviani E. Bernini V. (2020). Edible seaweeds and spirulina extracts for food application: in vitro and in situ evaluation of antimicrobial activity towards foodborne pathogenic bacteria. *Foods* 9 1–15. 10.3390/foods9101442 33053649 PMC7601287

[B36] Nidheesh T. Salim C. Rajini P. S. Suresh P. V. (2016). Antioxidant and neuroprotective potential of chitooligomers in *Caenorhabditis elegans* exposed to monocrotophos. *Carbohydr. Polym.* 135 138–144. 10.1016/j.carbpol.2015.08.055 26453861

[B37] Nuhu A. A. (2013). Spirulina (*Arthospira*): an important source of nutritional and medicinal compounds. *J. Mar. Sci.* 2013:325636. 10.1155/2013/325636

[B38] Ozdemir G. Karabay N. U. Dalay C. M. Pazarbasi B. (2004). Antibacterial activity of volatile component and various extracts of *Spirulina platensis*. *Phytoth. Res.* 18 754–757. 10.1002/ptr.1541 15478198

[B39] Palacios-Gorba C. Pina R. Tortajada-Girbés M. Jimenez-Belenguer A. Siguemoto E. Ferrús M. A. (2020). Caenorhabditis elegans as an *in vivo* model to assess fucoidan bioactivity preventing *Helicobacter pylori* infection. *Food Funct.* 11 4525–4534. 10.1039/D0FO00768D 32393934

[B40] Pankaj S. K. Wan Z. Keener K. M. (2018). Effects of cold plasma on food quality: a review. *Foods* 7:4. 10.3390/foods7010004 29301243 PMC5789267

[B41] Peixoto H. Roxo M. Krstin S. Röhrig T. Richling E. Wink M. (2016). An anthocyanin-rich extract of acai (*Euterpe precatoria* Mart.) increases stress resistance and retards aging-related markers in *Caenorhabditis elegans*. *J. Agric. Food Chem.* 64 1283–1290. 10.1021/acs.jafc.5b05812 26809379

[B42] Peixoto H. Roxo M. Röhring T. Riching E. Wang X. Wink M. (2017). Anti-aging and antioxidant potential of *Paullinia cupana* var. *sorbilis*: findings in *Caenorhabditis elegans* indicate a new utilization for roasted seeds of guarana. *Medicines* 4:61. 10.3390/medicines4030061 28930275 PMC5622396

[B43] Pérez-Andrés J. M. Álvarez C. Cullen P. J. Tiwari B. K. (2019). Effect of cold plasma on the techno-functional properties of animal protein food ingredients. *Innov. Food Sci. Emerg. Technol.* 58 1–7. 10.1016/j.ifset.2019.102205

[B44] Pina-Pérez M. C. Martinet D. Palacios-Gorba C. Ellert C. Beyrer M. (2020). Low-energy short-term cold atmospheric plasma: controlling the inactivation efficacy of bacterial spores in powders. *Food Res. Int.* 130 1–10. 10.1016/j.foodres.2019.108921 32156369

[B45] Pina-Pérez M. C. Rivas A. Martínez A. Rodrigo D. (2017). Antimicrobial potential of macro and microalgae against pathogenic and spoilage microorganisms in food. *Food Chem.* 235 34–44. 10.1016/j.foodchem.2017.05.033 28554644 PMC7131516

[B46] Pina-Pérez M. C. Rivas A. Martinez A. Rodrigo D. (2018). Effect of thermal treatment, microwave, and pulsed electric field processing on the antimicrobial potential of açaí (*Euterpe oleracea*), stevia (*Stevia rebaudiana* Bertoni), and ginseng (*Panax quinquefolius* L.) extracts. *Food Control* 90 98–104. 10.1016/j.foodcont.2018.02.022

[B47] Rodríguez Ó Gomes W. F. Rodrigues S. Fernandes F. A. (2017). Effect of indirect cold plasma treatment on cashew apple juice (*Anacardium occidentale* L.). *LWT Food Sci. Technol.* 84 457–463.

[B48] Salgueiro W. G. Goldani B. S. Peres T. V. Miranda-Vizuete A. Aschner M. Teixeirada Rocha J. B. (2017). Insights into the differential toxicological and antioxidant effects of 4-phenylchalcogenil-7-chloroquinolines in *Caenorhabditis elegans*. *Free Radic. Biol. Med.* 110 133–141. 10.1016/j.freeradbiomed.2017.05.020 28571752

[B49] Sanz-Puig M. Arana-Lozano A. Pina-Pérez M. C. Fernandez P. Martinez A. Rodrigo D. (2020). Occurrence of *Salmonella typhimurium* resistance under sublethal/repeated exposure to cauliflower infusion and infection effects on *Caernohabditis elegans* host test organism. *Food Sci. Technol. Int.* 26 151–159. 10.1177/1082013219873500 31544526

[B50] Sanz-Puig M. Lázaro E. Armero C. Alvares D. Martínez A. Rodrigo D. (2017). *S. typhimurium* virulence changes caused by exposure to different non-thermal preservation treatments using *C. elegans*. *Int. J. Food Microbiol.* 4 49–54. 10.1016/j.ijfoodmicro.2017.09.006 28963905

[B51] Sarangapani C. Keogh D. R. Dunne J. Bourke P. Cullen P. J. (2017). Characterisation of cold plasma treated beef and dairy lipids using spectroscopic and chromatographic methods. *Food Chem.* 235 324–333. 10.1016/j.foodchem.2017.05.016 28554643

[B52] Seghiri R. Kharbach M. Essamri A. (2019). Functional composition, nutritional properties and biological activities of Moroccan Spirulina microalga. *J. Food Quality* 2019 1–12. 10.1155/2019/3707219

[B53] Stiernagle T. (2006). *Maintenance of C. elegans The C. elegans Research Community, WormBook (eds).* 1–11. Available online at: http://www.wormbook.org/chapters/www_strainmaintain/strainmaintain.html (accessed January 10, 2022). 10.1895/wormbook.1.101.1 PMC4781397 18050451

[B54] ten Bosch L. Pfohl K. Avramidis G. Wieneke S. Viöl W. Karlovsky P. (2017). Plasma-based degradation of mycotoxins produced by *Fusarium*, *Aspergillus* and *Alternaria* species. *Toxins (Basel)* 9:97. 10.3390/toxins9030097 28287436 PMC5371852

[B55] Teshiba E. Miyahara K. Takeya H. (2016). Glucose-induced abnormal egg-laying rate in *Caenorhabditis elegans*. *Biosci. Biotechnol. Biochem.* 80 1436–1439. 10.1080/09168451.2016.1158634 26966894

[B56] Tolouie H. Mohammadifar M. A. Ghomi H. Hashemi M. (2018). Cold atmospheric plasma manipulation of proteins in food systems. *Crit. Rev. Food Sci. Nutr.* 58 2583–2597. 10.1080/10408398.2017.1335689 28613926

[B57] Whitehead J. C. (2016). “Chapter 3– The chemistry of cold plasma,” in *Cold Plasma in Food and Agriculture Fundamentals and Applications*, eds Misra N. N. Schlüter O. Cullen P. J. (Amsterdam: Academic Press), 53–81. 10.1016/B978-0-12-801365-6.00003-2

[B58] Wilson M. A. Hunt P. R. Wolkow C. A. (2010). “Using *Caenorhabditis elegans* to study bioactivities of natural products from small fruits,” in *Flavor and Health Benefits of Small Fruits*, eds Qian M. C. Rimando A. M. (Washington, DC: ACS Symposium Series), 227–238. 10.1021/bk-2010-1035.ch014

[B59] Winkler-Moser J. K. Hwang H. S. Kerr B. J. (2020). Changes in markers of lipid oxidation and thermal treatment in feed-grade fats and oils. *J. Sci. Food Agric.* 100 3328–3340. 10.1002/jsfa.10364 32112406

[B60] Ziuzina D. Misra N. N. (2016). “Chapter 9–Cold plasma for food safety,” in *Cold Plasma in Food and Agriculture Fundamentals and Applications*, eds Misra N. N. Schlüter O. Cullen P. J. (London: Academic Press), 223–252.

